# Recent Advances in Preparation and Application of BOPP Film for Energy Storage and Dielectric Capacitors

**DOI:** 10.3390/molecules30071596

**Published:** 2025-04-02

**Authors:** Kelei Zhang, Junlong Yao, Fangju Zhu, Yuan Gao, Yixi Gu, Yani Guo, Yimin Sun, Yu An

**Affiliations:** 1 Hubei Key Laboratory of Plasma Chemistry and Advanced Materials, School of Materials Science and Engineering, Wuhan Institute of Technology, Wuhan 430205, China; zncbm98@163.com (K.Z.); guoyani@wit.edu.cn (Y.G.); ymsun@wit.edu.cn (Y.S.); anyvbaba@163.com (Y.A.); 2 Zolia Quartz Stone Co., Ltd., Macheng 438300, China; 3 Analysis and Testing Center, Wuhan Institute of Technology, No. 206 Guanggu 1st Road, Wuhan 430205, China; 13006116506@163.com (Y.G.); guyixi@wit.edu.cn (Y.G.)

**Keywords:** polypropylene film, energy storage, dielectric capacitor, modification, stretching process

## Abstract

Energy storage polymers are critical to modern microelectronics, electric vehicles, and wearable devices. Capacitor energy storage devices are the focus of contemporary research, with film dielectric capacitors being the focus of mainstream research. Research on polymers—particularly polypropylene—has yielded numerous innovations, but their energy storage performance and breakdown resistance under extreme conditions remain unsatisfactory. Numerous reports have proposed various solutions, but systematic reviews, classifications, and investigations regarding the effects of processing on polypropylene films remain lacking. This study collects and organizes the latest research reports on dielectric-related polypropylene films with the aim of addressing this issue by providing a comprehensive review of the research on polypropylene thin film materials that exhibit high dielectric stability and high energy storage density under extreme conditions. These conditions include mixing and doping, surface modification, designing new molecular structures, and constructing multilayers. This study analyzes how polypropylene’s dielectric properties can be enhanced. It reviews the impacts of processing on the dielectric properties of biaxially oriented polypropylene and the underlying mechanisms. The paper is concluded with a summary of the current research progress and shortcomings in industrial production and performance, as well as discussions of future prospects. It offers valuable references for enhancing the dielectric properties of biaxially oriented polypropylene films and optimizing film processing.

## 1. Introduction

**Figure 1:**
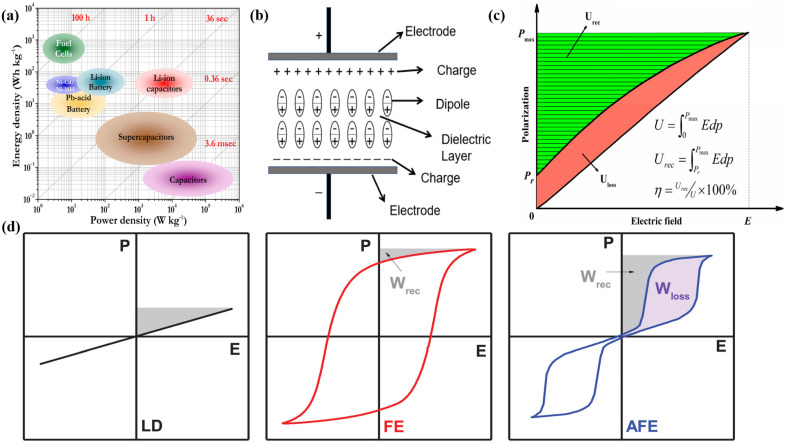
(**a**) Energy densities, power densities, and discharge rates of different energy storage methods [14]. Copyright 2014, *Chemical Reviews*. (**b**) Schematic diagram of the dielectric between two electrodes where the dipoles are aligned and displaced along the direction of the electrical field due to the applied electrical field [15]. Copyright 2024, *Batteries*. (**c**) Electrical hysteresis curve of the dielectric (*U* is the saturation energy density, *U_rec_* is the energy storage density, *U_loss_* is the loss energy density, *E* is the electrical field strength, *P_max_* is the saturation polarization, *P_r_* is the residual polarization, and *η* is the charge/discharge efficiency) [16]. Copyright 2022, *Journal of Materials Science: Materials in Electronics*. (**d**) Electrical hysteresis curves for linear dielectric (LD), ferroelectric (FE), and antiferroelectric (AFE), where *W_rec_* is the energy storage density and *W_loss_* is the loss energy density [17]. Copyright 2022, *Energy Storage Materials*.

The ongoing rise in high-power applications such as electric vehicles, microelectronics, and pulsed power systems is driving the need for a new generation of high-temperature dielectric materials for capacitive energy storage [1,2]. Polymer dielectrics are characterized by light weight, scalability, mechanical flexibility, high dielectric strength, and extreme reliability. In addition, polymer dielectric capacitors are more efficient and environmentally sustainable than traditional energy sources [3,4]. In comparison with energy storage devices such as supercapacitors, fuel cells, and lithium batteries, dielectric capacitors exhibit several advantageous properties, including low weight, enhanced processability, and rapid charging and discharging rates [5,6]. Polymer dielectric capacitors have the advantages of a high power density and ultra-fast charging and discharging rates in capacitive energy storage [7,8,9], as illustrated in Figure 1a. Dielectric capacitors utilize an electrical field to charge the disordered dipole inside the unpolarized dielectric, thus conforming it to the direction of the electrical field aligned with the direction of the electrical energy of the directional dipole to store the energy [10,11], as shown in Figure 1b. The directional electrical dipole’s recurrence of disorder releases stored electrical energy. The apparatus uses the polarization and depolarization of dielectric materials to charge and discharge capacitors [12]; this process is shown in Figure 1c, where the green area indicates energy storage, and the red area indicates energy loss. The energy density (*U_e_*) of the dielectric can be described by the equation $U_{e}=\int_{D_{r}}^{D_{m}}EdD$ [13], where E is the electrical field strength applied to the dielectric, and *D_m_* and *D_r_* are the maximum and residual potential shifts.

Polymer dielectric materials possess a high breakdown strength under both AC and DC, and exhibit low conductivity and loss performance characteristics [18]. At the same time, polymers offer advantageous properties such as ease of processing and good flexibility. Therefore, the use of polymers as dielectrics is aligned with the current flexibilization and miniaturization of electronic and electrical equipment [19]. When the dielectric is a linear dielectric (i.e., the polarization strength varies linearly with the electrical field strength, with an electrical hysteresis curve as shown in Figure 1d), its energy density equation can be simplified to $U_{e}=1/2\varepsilon_{0}\varepsilon_{r}E_{b}^{2}$ [20,21], where *ε_0_* and *ε_r_* are the dielectric constant of the vacuum and the relative dielectric constant of the material, respectively, and E_b_ is the breakdown strength of the material.

Polypropylene (PP) is a major dielectric material due to its superior chemical stability, high breakdown strength, low dielectric loss, and good processability [22,23]. Biaxially oriented polypropylene (BOPP) plays an instrumental role in the field of commercial film capacitors [24]. BOPP has unique advantages compared to other capacitor materials. For example, metalized BOPP makes a better capacitor electrode than metal oxides because it is lighter, smaller, flexible, and easy to process. It also has a fuse mechanism, making it safer [25]. However, due to the inherent limitations of PP, including its low relative dielectric constant and low energy storage density [26], it falls short of meeting the stringent requirements for device lightweighting and integration [27]. In particular, the dielectric properties of PP substantially decline at elevated temperatures (105 °C), resulting in a sudden drop in energy storage density in high-temperature environments. This can lead to capacitor failure or safety hazards [28]. BOPP’s incorporation into the film-forming process during electroweak interactions or defective pore fabrication may reduce the breakdown strength, hindering energy storage performance. This hinders a further reduction in the thickness of BOPP, constraining enhancement of the energy storage density in film capacitors [29]. At present, BOPP cannot be applied in specific scenarios involving high temperatures or high voltages [30]. It does not align with the current stage of development in the field of electrical equipment.

Although many advances have been made in the study of PP, many problems remain, such as the fact that the very low energy storage density of nonpolar PP limits the development of higher-capacity capacitors [31], and the dielectric stability under extreme conditions is still unsatisfactory [32]. In this paper, we mainly collect and organize studies on high-performance BOPP for dielectric energy storage published in recent years. Firstly, we present an overview of the endeavors by researchers to enhance the dielectric stability and energy storage density of PP. Subsequently, the current research progress on the impact of BOPP processing on its dielectric properties is reviewed, in order to provide a reference point for the advancement of BOPP applications in the domains of energy storage and capacitors. Finally, the current research progress and trends of BOPP are summarized, in addition to providing ideas and suggestions for the further development of BOPP in terms of dielectric stability, energy storage, and processing technology.

## 2. Polypropylene with High Energy Storage and Dielectric Stability

PP has a high breakdown strength and low dielectric constant under a normal working environment; however, under specific conditions, its performance can deteriorate significantly due to molecular chain slippage, potentially leading to failure in certain applications [26,33]. This poses a significant challenge to the effective use of PP in various types of electronic equipment. The absence of polar groups contributes to PP’s enhanced chemical stability and lower dielectric constant compared to other dielectric polymers. This defect hinders the enhancement of PP’s energy storage density, impeding improvements in capacitors’ energy storage characteristics [34,35,36]. The future directions of electrical and electronic equipment are miniaturization and being lightweight. Thus, researchers are working to improve PP’s dielectric stability and energy storage properties to enable its operation under extreme conditions.

### 2.1. Co-Dopant

#### 2.1.1. Incorporation of Inorganic Filler

Nanofillers doped into PP reduce the spatial charge magnitude in PP. This effect is more pronounced at higher nanofiller loading levels, due to the fact that the addition of the fillers inhibits the injection of charge into the PP [37,38]. Thus, doped inorganic fillers are being used to enhance the dielectric and energy storage characteristics of PP [39]. Inorganic filler doping also has a better effect in preventing the thermal aging of PP and thus improving its dielectric stability. Magnesium aluminate (MgAl_2_O_4_), calcium carbonate (CaCO_3_), and surface-modified calcium carbonate (CaCO_3_T) nanofillers have been added to PP, and the results proved that the addition of these inorganic fillers effectively prevented the decay of the breakdown strength and relative dielectric constant of PP at high temperatures. This is because the inorganic fillers not only confer the characteristic of a high dielectric constant, but also serve as a nucleating agent; the internal original large spherical crystals are refined and the defective areas are reduced because the molecular chains’ movement is restricted, enhancing the insulating properties [40]. However, adding inorganic fillers directly can lead to defects at the interface, due to nanoparticle agglomeration and its compatibility with PP. Thus, modifying the surfaces of nanoparticles can improve the composite material’s temperature resistance [41].

**Figure 2:**
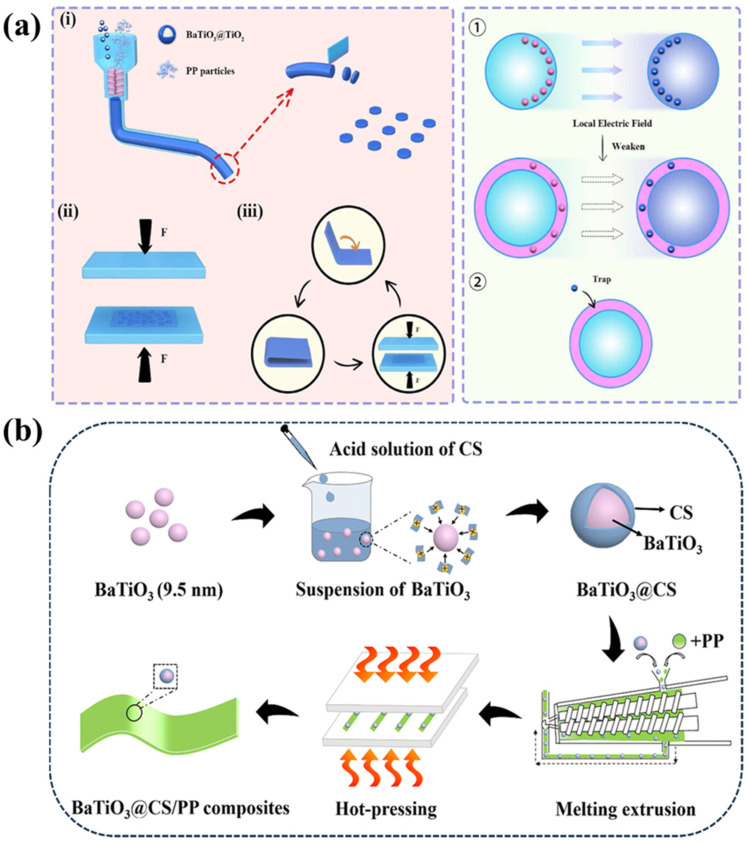
(**a**) Design and preparation of BaTiO_3_@TiO_2_/PP composites; i: BaTiO_3_@TiO_2_ the preparation process; ii: BaTiO_3_@TiO_2_ join PP and heat press it; iii: BaTiO_3_@TiO_2_/PP composites the preparation process; ①: BaTiO_3_@TiO_2_ generate electron traps under the action of an electric field; ②: Carriers enter electron traps [44]. Copyright 2022, *Materials Today Energy*. (**b**) Fabrication scheme for BaTiO_3_@CS and BaTiO_3_@CS/PP composites [46]. Copyright 2024, *Journal of Materials Chemistry C*.

Barium titanate (BaTiO_3_)—a ferroelectric ceramic material with a high dielectric constant—plays a significant role in enhancing the dielectric constant of PP [42]. In addition, its ability to induce strong interfacial polarization can increase the interfacial polarization of the composites, while nanoparticles can act as electron traps to inhibit the carrier motion and hinder the growth of “electrical trees”, thus enhancing the breakdown strength. However, when BaTiO_3_ and PP are blended, a serious electrical field distortion occurs at the interface, which triggers the electrical breakdown phenomenon. In addition, the high surface energy of the nanoparticles generates a serious agglomeration phenomenon, which is not conducive to improving the material’s properties and can easily introduce defects; thus, nanofillers often need to be modified to reduce their surface energy to reduce agglomeration. For example, upon using the silane coupling agent KH560 (3-glycidoxypropyltrimethoxysilane) as a modifier and grafting it onto BaTiO_3_ to reduce agglomeration, the performance of the prepared film was significantly improved, and its energy storage density was enhanced from 1.49 J cm^−3^ to 2.21 J cm^−3^ [43]. In addition, preparing titanium dioxide-coated barium titanate (BaTiO_3_@TiO_2_) particles with a core–shell structure increased the energy storage density of PP to 5.58 J cm^−3^ at 580 MV m^−1^, while still maintaining a 99.3% charge/discharge efficiency; the preparation process is shown in Figure 2a [44]. The surface energy was reduced through the amination of Ti_0.87_O_2_ modified with the silane coupling agent KH550 (γ-aminopropyl triethoxysilane) before doping with PP. The addition of fillers increased the capture carrier traps and also inhibited the formation of electrical trees, thus reducing the chance of electrical breakdown occurring. Dielectric constants up to 3.27 were achieved, which is 1.5 times higher than normal PP [45]. Compared with using an inorganic core–shell structure, using chitosan (CS) as the outer shell of the core–shell structure is more advantageous in terms of compatibility; the composite material preparation is shown in Figure 2b. Adding trace amounts of this type of core–shell structure greatly improved the energy storage density, which was 1.77 J cm^−3^ for pure PP vs. 4.76 J cm^−3^ when this was added, in addition to maintaining a charge/discharge efficiency of 93.9%. It even increased the breakdown strength up to 435 MV m^−1^ at 105 °C, compared to the 372 MV m^−1^ of pure PP [46]. The core–shell structure can give the composite dielectric a larger interfacial polarization, while the CS in the outer layer has a higher electron affinity, which introduces more deep traps that can trap free charges and inhibit the rapid development of an electrical tree. BaTiO_3_ provides an extra layer of buffered CS structure over PP; it restricts the carrier migration between the two and alleviates the leakage current phenomenon.

Incorporating inorganic fillers into polymeric materials improves the dielectric constant and introduces charge traps that can prevent spatial charge buildup. However, this integration can lead to challenges like agglomeration and poor compatibility due to the high surface energy of the fillers, potentially resulting in defects that reduce the polymer’s electrical insulation performance. There is an ongoing need to enhance the doping methodology for inorganic fillers, building on existing research. This enhancement should ensure the uniform dispersion of fillers within PP while investigating the characteristics of these introduced traps. This will facilitate the comprehensive utilization of their influence on the material’s dielectric properties.

#### 2.1.2. Blending with Organics

**Figure 3:**
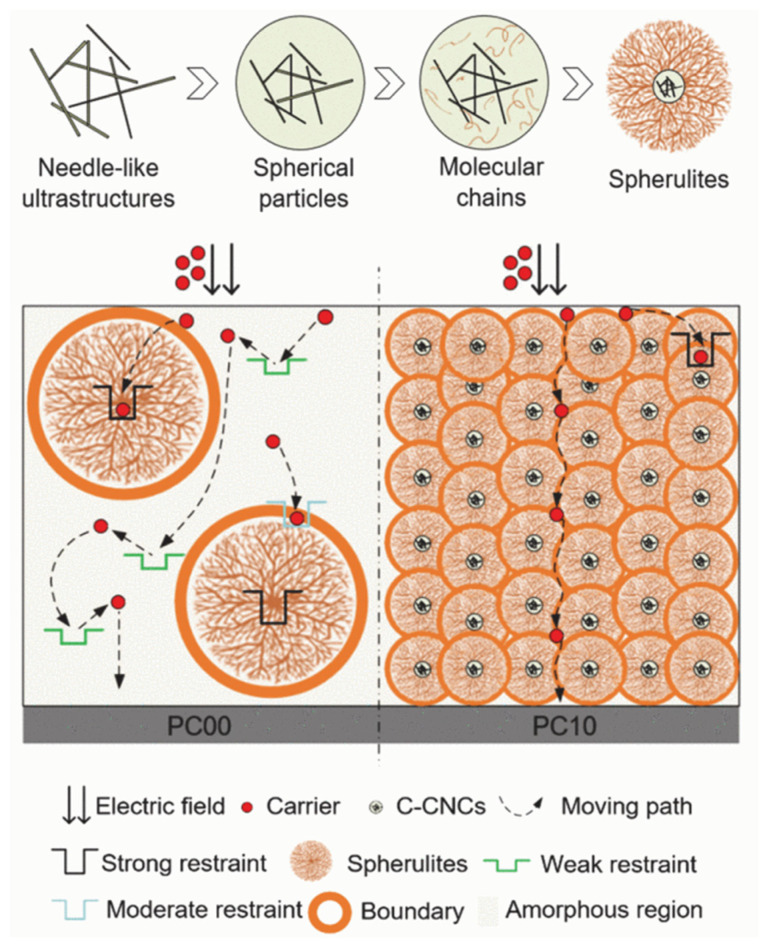
Schematic diagram of carrier transport in PC00 and PC10, the content of PC00 doped with C-CNCs is 0%, the content of PC10 doped with C-CNCs is 0.001% [49]. Copyright 2021, *IEEE Transactions on Dielectrics and Electrical Insulation*.

The addition of organics is more conducive to the preparation of PP composite films with excellent properties because it avoids the internal defects and agglomerations produced by the addition of inorganic fillers. The high-temperature dielectric properties of PP can be effectively improved by choosing to co-blend a more compatible cyclic olefin copolymer (COC) into PP and taking advantage of its excellent mechanical properties and high heat resistance. The breakdown strength at 105 °C is up to 358.9 MV m^−1^, marking a 17.17% increase, and the energy storage density is also increased from 0.92 J cm^−3^ to 1.38 J cm^−3^. The ring structure of COC can effectively inhibit the movement of PP’s molecular chains, preventing carriers from obtaining energy in the free volume and also making the internal crystalline region more dense, thereby reducing defects; this means that the conductivity loss at high temperatures is reduced, meaning that the breakdown strength and energy storage density are improved [47]. When dielectric capacitors operate in high magnetic field environments such as electromagnetic launch (EML) systems, the performance of capacitors with BOPP as the dielectric is degraded due to the magnetic field, which affects the safety and reliability of EML; blending aromatic compounds with PP can effectively improve the PP’s ability to withstand high-magnetic-field environments. Under a magnetic field strength of 12 T, the modified PP has an energy storage density of 2.72 J cm^−3^, compared to that of the modified PP of 2.07 J cm^−3^. This is due to the fact that the benzene ring of the aromatic compound has a more stable structure and also builds low-energy regions inside the PP; these low-energy regions effectively reduce the carrier mobility during charge transport, so the resistance and electrical insulation of the material are improved [48]. Metallized film capacitors (MFCs) are generally rated to operate at 85 °C, while the long-time operating temperature of PP generally needs to be lower than 80 °C. When the operating temperature rises, the molecular chains fracture, the defective region dramatically increases the carrier mobility, and the material’s insulating property decreases. The crystallization is promoted by micro-co-mixing carboxylated cellulose nanocrystals (C-CNCs). This makes the internal crystalline landscape denser and more complete, and the carrier transport process is shown in Figure 3. This allows for the maintenance of excellent breakdown performance at 85 °C [49].

The decrease in PP’s performance at high temperatures is due in part to the presence of internal spherical crystals with a looser structure and larger mean free range, in addition to the movement of molecular chains which leads to potential weakness. For example, long-chain branched polypropylene (LCBPP) can increase the entanglement of molecular chains, limiting the movement of those chains and preventing defective regions at high temperatures. At the same time, due to the principle of non-homogeneous phase nucleation, LCBPP also increases the number of spherical crystals and their refinement and density, reducing the weakness of the film’s internal region; the breakdown strength at 105 °C is increased by 16.2% compared to that of the PP [50]. The addition of an appropriate amount of nucleating agent on top of this also contributes to improving the dielectric properties of PP at high temperatures, with the breakdown strength at 125 °C reaching 521.5 MV m^−1^, marking a 26.3% increase, and the energy storage density reaching 2.79 J cm^−3^, for a 66.1% increase. This is attributed to the addition of nucleating agents and LCBPP making the spherical crystal structure more dense, meaning that carriers cannot be transported in the crystalline region, while, at the same time, the addition of LCBPP increases the entanglement of molecular chains, increasing the intermolecular forces such that the transport of carriers in the amorphous region also becomes more difficult [51]. Different nucleating agents have different effects on the internal crystalline structure of PP, while the dielectric and temperature resistance properties of the materials are also affected differently [33]. At high temperatures, the molecular chains of PP undergo partial decomposition, and the free radicals generated after this decomposition participate in further decomposition, which leads to an intensification of molecular chain decomposition. The addition of antioxidants can capture the free radicals generated in the molecular chains, thus slowing down their decomposition and improving the temperature resistance of PP [52].

**Figure 4:**
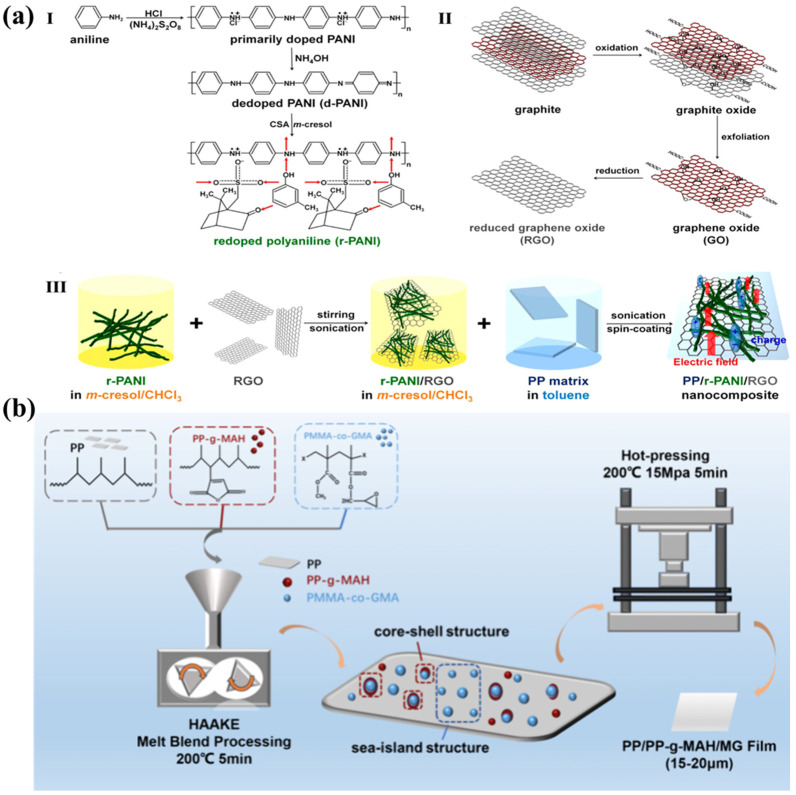
(**a**) Process flow for the preparation of (**I**) r-PANI nanofibers, (**II**) RGO sheets, and (**III**) PP/r-PANI/RGO nanocomposites [53]. Copyright 2015, *ACS Applied Materials & Interfaces*. (**b**) Process flow for the preparation of PP/PP-g-MAH and PP/PP-g-MAH/MG ternary composite membranes [54]. Copyright 2024, *ACS Applied Polymer Materials*.

Compared with inorganic fillers, polyaniline (PANI)—a highly dielectric polymer—with better compatibility with PP, and the dielectric properties of modified PP are also superior to those of PP modified with inorganic fillers. A composite prepared through the ternary blending of reduced graphene oxide (RGO), PANI, and PP has an energy storage density of up to 12.6 J cm^−3^ and a breakdown strength of 586 MV m^−1^; the process is shown in Figure 4a. This is attributed to the interface effect generated by the face-to-face stacking of PANI and RGO, which enhances the charge transfer ability of PANI, in addition to their high and uniform dispersion in PP. RGO and PANI in the composite material form an extensive microcapacitive structure with deep traps, resulting in more charge traps and a higher spatial charge density, thereby increasing the energy storage density [53]. Similarly, a methyl methacrylate–methacrylic acid glycidyl ester (MG) copolymer was bridged with polypropylene-grafted maleic anhydride (PP-g-MAH) to form an “island phase”-like structure and then integrated with the matrix PP to form an “island” structure of all-organic film. The preparation process is shown in Figure 4b PP-g-MAH as a polar compatibilizer, which not only prevents the agglomeration of MGs by lowering the interfacial tension, but also improves their affinity, reduces interfacial defects, and thus enhances the breakdown strength; at the same time, its own polar groups and the introduction of a large number of deep traps are also conducive to improving the dielectric constant. The formation of multiple interfaces within the composite film is conducive to promoting spatial charge polarization at the interfaces, thus leading to an increase in the dielectric constant. MGs with stronger charge affinity can trap more carriers. Enhancing the dielectric properties and breakdown strength inevitably enhances the energy storage density; the energy storage density of the composite film is 5.87 J cm^−3^, which is 280% of that of PP, at 600 MV m^−1^, while the charging and discharging efficiencies are maintained at 96% [54]. As a semi-crystalline polymer, PP has a total of five different crystalline phase structures—α, β, γ, δ, and quasi-hexagonal—with the α phase being the most stable [55,56]. However, the relatively rod-like β crystals have superior breakdown and energy storage properties [57]. The effect of crystallinity on PP can be harnessed to modify PP in a new way; for example, co-mingling the β-nucleating agent WBG II induced the generation of β-crystals, which improved the crystallinity and enhanced the interfacial polarization; the β-crystals, with their special lamellar crystalline structure, also introduced a deep trap charge inhibition of charge transport in the crystalline region, and the more regular structure also enhanced the electrical breakdown performance. The dielectric properties of PP were substantially improved by this method, and the energy storage density reached 7.05 J cm^−3^ at a field strength of 581.6 MV m^−1^. Moreover, the films also possessed long-term cycling stability, with charging and discharging efficiencies over 96% after 50,000 ultra-high cycling iterations [58]. Different nucleating agents confer different modification effects [59]. Improving the crystalline structure effectively reduces the defective regions and enhances the performance and stability of PP.

Blending is a relatively simple modification, and it can bring the advantages of both blends into full play, but it also brings the disadvantages of each blend, and the compatibility problem between the polymers still exists. For this reason, it is necessary to continue exploring and improving the selection of blends and the method of blending.

#### 2.1.3. Multi-Blending

**Figure 5:**
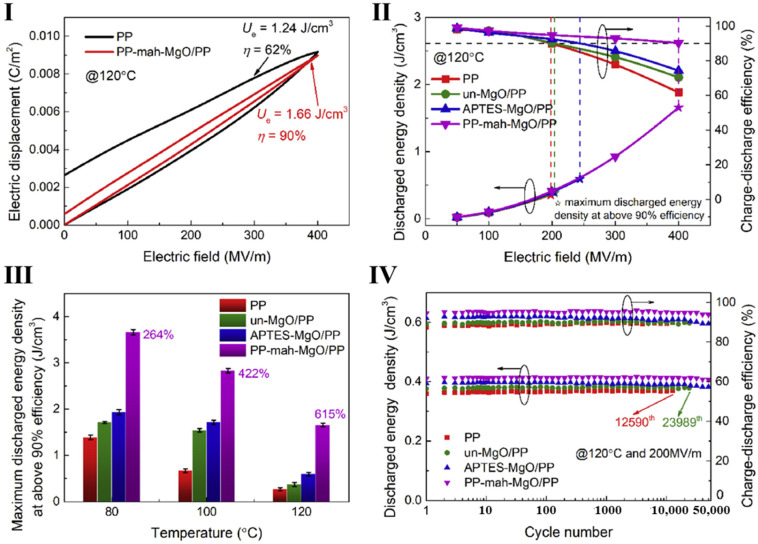
High-temperature energy storage performance of PP and PP nanocomposites. APTES-MgO is the amino-functionalized MgO nanoparticles, un-MgO is the MgO nanoparticles. (**I**) Polarization curves of pure PP and PP-mah-MgO/PP at 400 MV m^−1^ at 120; (**II**) charge/discharge efficiency and discharge energy density at 120 °C; (**III**) maximum energy storage density achieved at more than 90% charge/discharge efficiency measured at 80 °C and 120 °C; (**IV**) cyclic charge/discharge performance at 200 MV m^−1^ and 120 °C [62]. Copyright 2020, Energy Storage Materials.

Compared with pure inorganic doping or organic blending, the rational addition of both blends is a unique option. By preparing a ternary composite system with PP, nano-ZrO_2_ (zirconium dioxide), and PP-g-MAH, the effective electric field strength is maintained at 20 MV m^−1^, and the energy storage density is 17.3% higher than that of pure PP, which is attributed to the hydrogen bonding system introduced into the construct by co-doping, as well as a large number of deep traps, which effectively enhance the compatibility between the substrate and the filler and reduce the free volume and spatial charge accumulation, leading to an increase in the DC breakdown strength. At the same time, the enhanced intermolecular forces hinder the ionic hopping, thus limiting the ionic conduction at high field strengths, reducing the dielectric loss [60]. The incorporation of ZrO_2_ confers more than just a high dielectric constant; it introduces more deep traps. The addition of PP-g-MAH not only enhanced the polarity, improving the compatibility between ZrO_2_ and PP, but also formed a network structure together with ZrO_2_; the network connection points restricted the movement of molecular chains, so electron transport was inhibited. A 30.2% increase in energy storage density was observed in comparison with PP [61]. After the addition of ceramic particles MgO (magnesium oxide) and PP-g-MAH—PP-mah-MgO, the film maintained a charge/discharge efficiency more than 90% at 120 °C and a 400 MV m^−1^ field strength, while the energy storage density was still 615% higher at 1.66 J cm^−3^ than that of pure PP, as shown in Figure 5 [62]. In addition, changing the type of compatibilizer can also have a great impact on the film; for example, replacing the compatibilizer with polypropylene-grafted acrylic acid (PP-g-AA) to make a modified composite film at 446 MV m^−1^ and 120 °C led to an energy storage density of 2.28 J cm^−3^, which is 670% of that of PP [63]. This indicates that the effect of PP-g-AA on the properties of the composite film is different from that of PP-g-MAH [64].

Inorganic and organic dopant blends can significantly improve the dielectric and mechanical properties of PP, but these additives cannot produce a uniform dispersion in PP. These unevenly dispersed fillers will bring about defects in the electrical insulation properties and mechanical properties. For this reason, it is necessary to continue exploring the selection of dopants in the blend, and modifying the blend in advance to enhance its compatibility with PP may be a new interesting idea.

### 2.2. Surface Modification

#### 2.2.1. Surface Coating

**Figure 6:**
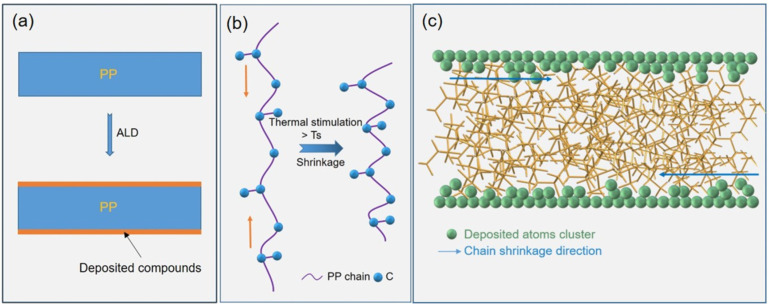
Experimental principle and microstructure characterization. (**a**) Schematic diagram of ALD deposition. (**b**) Schematic of molecular chains shrinking from thermal stimulation. (**c**) Schematic of the ALD inorganic layer anchoring the chain from moving [66]. Copyright 2021, *Nano Express*.

**Figure 7:**
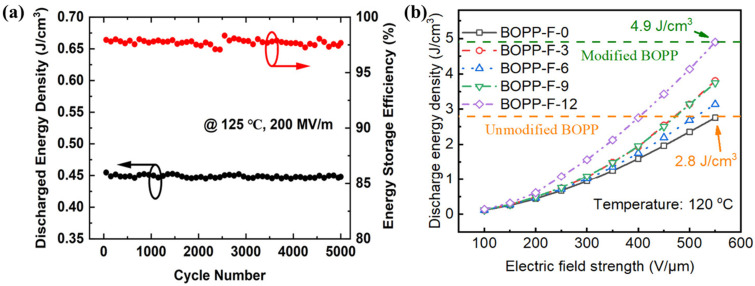
(**a**) Discharge energy density and energy storage efficiency of Al_2_O_3_/BOPP/Al_2_O_3_ thin films at 200 MV m^−1^ at 125 °C vs. number of cycles [70]. Copyright 2022, *ACS Applied Energy Materials*. (**b**) Modified BOPP electric field versus energy storage density. (The number represents the pressure spray treatment time, and the unit is s) [65] Copyright 2024, *ACS Applied Polymer Materials*.

Compared to the use of inorganic fillers to improve the properties of PP, surface modification has a lower impact on compression resistance and flexibility and is more conducive to the production of thin films, in addition to being a relatively simple and fast process. The thinning of PP will lead to a deterioration in breakdown strength and high temperature resistance [65]. Thus, the surface of BOPP was coated with a layer of Al_2_O_3_ through atomic layer deposition (ALD) to limit the movement of molecular chains on the surface of the film and to maintain the orientation of the internal crystalline pattern, as shown in Figure 6. The experiments demonstrated that the dimensional stability of the films was substantially improved. As the surface is coated with Al_2_O_3_, the atoms and molecular chains are tightly bound to minimize surface defects, substantially limiting the movement of the chains, charge transport, and the generation of breakdown channels, thereby enhancing the breakdown strength at high temperatures. The breakdown strength of the coated BOPP at 140 °C was reduced by only 24% up to 490 MV m^−1^, compared to a 35% reduction in the breakdown strength of the PP film [66]. In addition to ALD, magnetron sputtering is a common surface modification method; sputtering nanoceramic particles that are broadband, that have high thermal conductivity, and that are highly dielectric onto the surface of BOPP can enhance its temperature resistance and dielectric properties [67,68]. Placing a broadband coating on the surface of polymer films is an effective method for inhibiting the charge injection at metal electrodes and for improving the high-temperature energy storage performance. The magnetron sputtering of an aluminum nitride (AlN) coating on both sides of the BOPP built a sandwich structure, effectively improving the film’s high-temperature energy storage performance. This was due to the wide bandgap of the electrodeless coating forming an interfacial barrier that protected the film, which significantly inhibited the injection of electrons and charge transport; ultimately, the film could be maintained at 125 °C, with charge and discharge efficiencies more than 90% and an energy storage density of 1.5 J cm^−3^ [69]. In addition, upon introducing Al_2_O_3_ onto both sides of the BOPP, the dielectric constant of the composite film was stably maintained above 2.2 at 125 °C, while the number of charge-discharge cycles was above 5000, as shown in Figure 7a. The energy storage density of the modified BOPP at 125 °Cand 450 MV m^−1^ was maintained at 2.15 J cm^−3^, which is 36.1% higher than that achieved with pure PP [70]. Inevitably, some damage occurs during bi-directional stretching; this damage loosens the molecular chains, making them more mobile [60], thus accelerating the decomposition of the chain segments leading to the degradation of the insulating properties of the BOPP, and thus the energy storage density repairing the surface of the BOPP prior to its use is an option for addressing this issue. Repairing the defects on the surface of BOPP with a modifying spray containing polytetrafluoroethylene (PTFE) and n-butyl titanate increased the high-temperature breakdown field strength by 28.3%. The energy storage density at 120 °C was also significantly increased, up to 4.9 J cm^−3^, as shown in Figure 7b [71]. The polar polymers and additives within the coating also simultaneously improved the dielectric constant of the composite film.

The physical coating of BOPP can effectively improve the movement of molecular chains on the surface, inhibit the injection of charge, and repair the defects on the surface of the BOPP. However, there are some drawbacks: the coating method may introduce new defects or even break the film. In addition, due to the nonpolar nature of PP, its low surface energy cannot produce a strong bond with the coating, leading to safety problems during use, so many factors remained to be explored for this type of modification.

#### 2.2.2. Surface Chemical Modification

**Figure 8:**
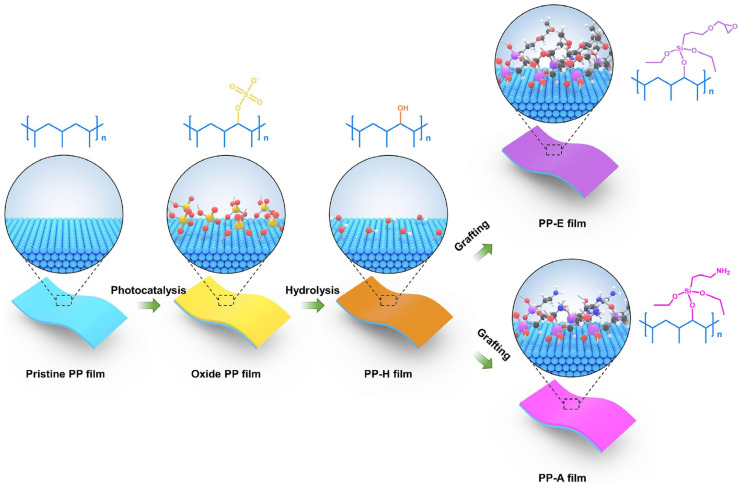
Schematic diagram for the preparation of surface-modified samples [76]. Copyright 2025, *Chemical Engineering Journal*.

Chemical modification is more reliable, durable, and convenient than physical coating. The process of aging in BOPP can be effectively suppressed by surface phosphorylation. This is due to the generation of more stable carboxylic acids on the surface of BOPP under the action of electrothermal heat. This can effectively reduce the decomposition rate of the BOPP chain segments. The dielectric stability of BOPP at high temperatures is thus ensured [72]. E-beam irradiation technology improves the structure of polymers. It changes the polarity and defects on the surface, and the radiation can also be transmitted to the interior of the polymer, exerting a certain degree of influence on the polymer’s overall structure, crystalline shape, and so on [73,74]. Generally speaking, due to the action of radiation, the internal molecular chains become fractured, crosslinked, oxidized, etc., so the original long straight chain structure of PP is transformed into a star-shaped long-chain branched structure; at the same time, the electron beam can improve the polarity of the PP film, due to the radiation generating free radicals that react with oxygen to produce hydroxyl, carbonyl, and other polar groups, as well as the production of a number of chemical defects in the electron traps, which enhance the electron trapping, inhibiting charge transfer. These phenomena lead to the relative dielectric permittivity of the film, which results in a number of defects. These enhance the relative dielectric constant (3.98) and electrical breakdown strength (430 MV m^−1^) of the films. In addition, due to these improved properties, the energy storage density was enhanced to 279% of that of PP, at 3.6 J cm^−3^ [75]. The film modified through electron beam irradiation still had charge/discharge efficiency over 95%. The surface was hydroxylated by the UV radiation technique and then grafted with the silane coupling agent KH550 or KH560 to obtain the surface amino-modified PP and epoxy-modified PP. The modification process is shown in Figure 8. The modified PP had an enhanced dielectric constant due to the introduction of polar functional groups to enhance the dipole migration and polarization strength. Also, the breakdown strength at 120 °C was increased from 522.3 MV m^−1^ to 643.1 MV m^−1^,and the energy storage density was enhanced by 97.4% to 3.08 J cm^−3^ [76]. This was mainly due to the higher deep trap density and energy levels of the modified PP. These deep trap layers can trap more carriers from the electrodes, thus suppressing carrier migration within the dielectric, improving the breakdown strength.

Physical coating methods for chemical modification have been shown to effectively circumvent the agglomeration problem, but they also damage the BOPP surface and cause film breakage. To address this issue, the modification process must be enhanced, paying particular attention to detail. Enhancing the quality of BOPP is identified as the pivotal factor in minimizing accidents.

### 2.3. Design of Molecular Structures

#### 2.3.1. Construction of Crosslinked Structures

**Figure 9:**
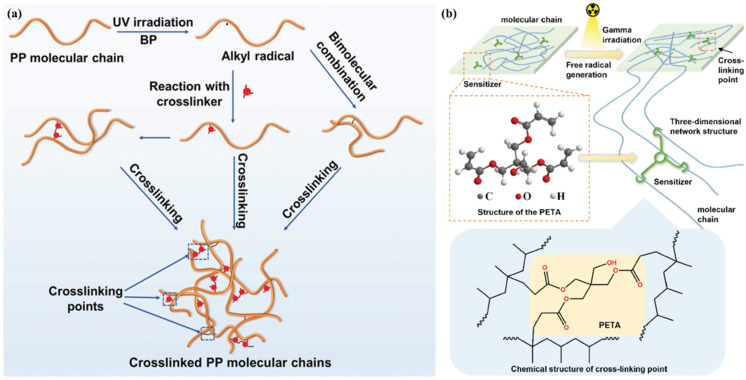
(**a**) Schematic representation of UV-initiated crosslinking reaction of PP molecular chains [28]. Copyright 2024, *IEEE Transactions on Dielectrics and Electrical Insulation*. (**b**) Schematic representation of PP crosslinking under γ-ray irradiation [81]. Copyright 2024, *IEEE Transactions on Dielectrics and Electrical Insulation*.

Crosslinking is a chemical reaction between molecular chains that forms a reliable network structure [77]. There are two main ways to form crosslinks: one is chemical crosslinking by forming chemical bonds in the molecules, and the other is by irradiation to induce the formation of chemical bonds between the molecular chains to achieve crosslinking [78]. The severe degradation of PP’s properties at high temperatures is mainly due to the movement of linear chain segments at high temperatures, resulting in the destruction of crystals within the crystalline regions, and in the amorphous regions where localized relaxation occurs, resulting in a decrease in breakdown strength [79]. The proper crosslinking of the molecular chains within the PP can effectively prevent the movement of the chains; such a crosslinking reaction is shown in Figure 9a. This reaction can also be used to introduce deep traps to trap the free charge and limit the migration of carriers. The PP is crosslinked and entangled using photoinitiators and crosslinkers under UV irradiation. The breakdown strength of the material is as high as 569.4 MV m^−1^, compared to 490.1 MV m^−1^ for pure PP, when operating at a stable 115 °C, effectively preventing the degradation of PP at high temperatures [80]. The presence of crosslinking sites in the crosslinking structure constituted by the molecular chains can also act as a potential trap.

Compared with UV irradiation, γ-rays have stronger penetration and can effectively reduce the introduction of impurities without the need for additives [82]. The thermal stability of linear PP molecular chains is poor, while the internal residual impurities also exacerbate the decomposition of the molecular chain at high temperatures; these factors increase the carrier concentration and mobility, while the PP molecular chains’ simple structure cannot effectively capture the carrier ability, resulting in a significant increase in the loss of electrical conductivity, and the high temperature resistance performance is poor [81]. γ-rays cause the amorphous region of PP to crosslink and form a 3D mesh structure, as shown in Figure 9b, strengthening the intermolecular forces and limiting the movement of the molecular chain segments, while the network structure of the crosslinking points can also play a role, forming deep traps that limit the movement of the carriers, which reduces the conduction loss and thereby enhances the breakdown strength. A breakdown strength of 542.3 MV m^−1^ at 125 °C was observed [83]. The crosslinked structure can effectively prevent the movement of molecular chains within the molecule; using butyl styrene (BSt) as a crosslinking agent successfully increased the energy storage density of PP to more than 5 J cm^−3^, which is twice as much as that of BOPP under the same conditions [84]. This is due to the fact that the crosslinked structure at a high field strength restricts the movement of the molecular chains, thereby limiting the movement of carriers inside, and thus enhancing the breakdown strength and energy storage density.

This study explored crosslinking molecular chains to enhance PP’s dielectric properties, but this approach is still in its exploratory stage due to the complexity of the structural relationships between chain segments and molecules. To further the field, it is imperative to explore methodologies with which to effectively utilize the deep traps on the molecular chain to capture charge, employ the crosslinked network structure to enhance the restriction of molecular chain movement, thereby improving high-temperature resistance and facilitating the effective formation of a crosslinked structure through molecular bonds.

#### 2.3.2. Grafting Organic Functional Groups

**Figure 10:**
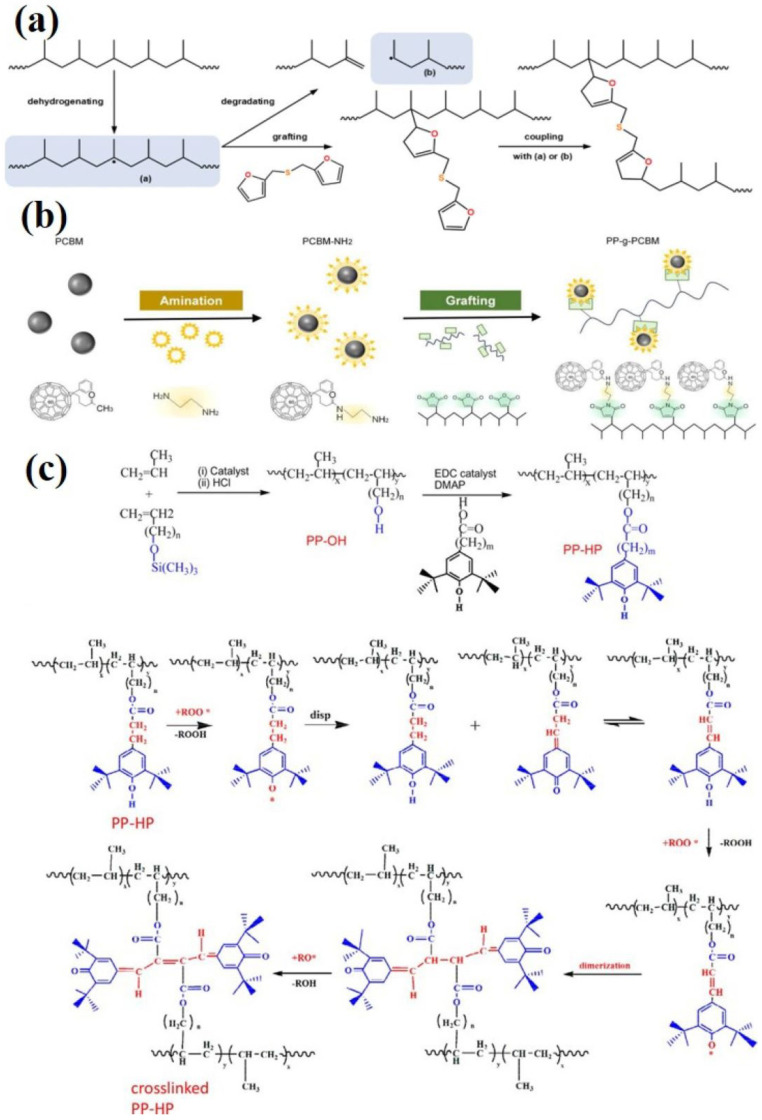
(**a**) Mechanism of FS grafting to PP main chain reaction [90]. Copyright 2024, *IEEE Transactions on Dielectrics and Electrical Insulation*. (**b**) Schematic of the surface amination and semiconductor grafting process of PCBM [91]. Copyright 2023, *Materials Today Energy*. (**c**) HP grafting to PP and reaction cross-linking mechanism; *represents free radicals [94]. Copyright 2020, *ACS Applied Materials & Interfaces*.

Redesigning and adjusting the molecular chain structure is the most efficient way to improve the properties of polymer dielectric films, which, unlike modification by adding inorganic fillers, can effectively prevent the generation of defects at the interface between the matrix and nanoparticles due to incompatibility [85,86]; optimizing the design and modulating the microstructure at the molecular level is beneficial for synergistically enhancing the mechanical, thermodynamic, and electrical properties. Many studies have used molecular structure modulation as an entry point to improve the temperature resistance of PP by changing its molecular chain structure. Graft modification is used to introduce polar functional groups or specific compounds into the molecular chains of the polymer, which induces crosslinking or entanglement among the chains, thus restricting their movement or introducing a deep-trapped trapping charge, thus weakening the interfacial electric field, lowering the rate of charge injection, effectively inhibiting the accumulation of spatial charge, and ultimately improving the electrical insulating properties of the polymer [87]. For example, methyl methacrylate (MMA) was branched onto the main linkage of PP through a water–solid phase suspension reaction, which ultimately enhanced the energy storage density from 1.35 J cm^−3^ for pure PP to 1.59 J cm^−3^ at a temperature of 125 °C, and the breakdown strength was enhanced from 537 MV m^−1^ to 764 MV m^−1^. This was attributed to the fact that the polar MMA moiety has a stronger electron affinity to capture more spatial charge, while the introduction of a large number of deep traps can effectively inhibit the loss at high temperatures, improving its dielectric properties at high temperatures [88]. Itaconic anhydride (ITA) is a polar molecule similar to maleic anhydride (MAH). The grafted PP with high electron affinity can capture the transported electrons through electrostatic attraction, thus introducing more deep traps in the PP, limiting the carrier migration at high temperatures; this means that it retains excellent dielectric properties at 120 °C. The polar ITA can also enhance the dielectric constant of the PP [89]. Due to the poor thermal stability of the amorphous region of PP and the weak intermolecular forces, the slip and local chain relaxation of the molecular chains in the amorphous region at elevated temperatures lead to an increase in the free volume and the average free range of the carriers, which leads to a decrease in the breakdown strength of PP. Furfuryl sulfide (FS) was grafted onto the PP main chain for long-chain branching, as shown in Figure 10a, to increase the entanglement and friction between molecular chains to prevent molecular chain slippage from hindering the free movement of the carriers, and to achieve the purpose of enhancing the thermal stability. The breakdown strength at 125 °C was increased by 17.45% to reach 490.5 MV m^−1^, and the storage density was also increased from 1.69 J cm^−3^ to 2.48 J cm^−3^ [90]. The organic semiconductor [6,6]-phenyl-C61-butyric acid isomethyl ester (PCBM) is more compatible with PP, and it can also introduce deeper trap sites inside, enhance the internal molecular forces, change the crystal structure of PP, and help to inhibit charge transfer; thus, the stability at high temperatures can be enhanced by grafting aminoclonalized PCBM to the PP backbone, as shown in Figure 10b. The dielectric constant of the PP film was successfully increased from 2.15 to 2.36 at 120 °C, and the dielectric loss was decreased by 68.5% to only 0.0029 compared with that of the PP film. The breakdown strength of the modified film reached more than 600 MV m^−1^, and the charge/discharge efficiency was maintained at more than 90%, while the energy storage density was increased by 683.62% to 1.59 J cm^−3^. This was attributed to the reduced conduction loss of the thin film preventing decreases in the breakdown strength and energy storage performance of the material at high temperatures [91]. At high temperatures, PP causes a significant increase in dielectric loss due to thermal degradation [92,93]. Although the free radicals generated by thermal degradation can increase the free charge, the small molecules generated by fracture also produce plasticization and enhanced charge mobility. Thus, the most direct way to improve thermal stability and limit decomposition by free radicals is to incorporate antioxidants. Connecting the crosslinkable antioxidant hindered phenol (HP) to PP not only provides an antioxidant effect that scavenges free radicals at high temperatures, but also induces a certain degree of crosslinking that achieves molecular linkage entanglement, impeding the movement of the molecular chains; the reaction crosslinking process is shown in Figure 10c. The film was treated at 170 °C for 24 h and was still able to maintain a breakdown strength of 350 MV m^−1^, which shows a significant improvement in the dielectric reliability of the film [94]. Similar to the grafting approach, the direct attachment of functional groups to the PP main chain during copolymerization is also an interesting direction of modification. For example, a carbazole was copolymerized directly with propylene to synthesize a special PP containing carbazole in the main chain, and then it was crosslinked internally with UV light to form a network structure. The modified PP has a high relative dielectric constant (>3) and improved thermal stability, enabling stable operation at high temperatures [95].

**Figure 11:**
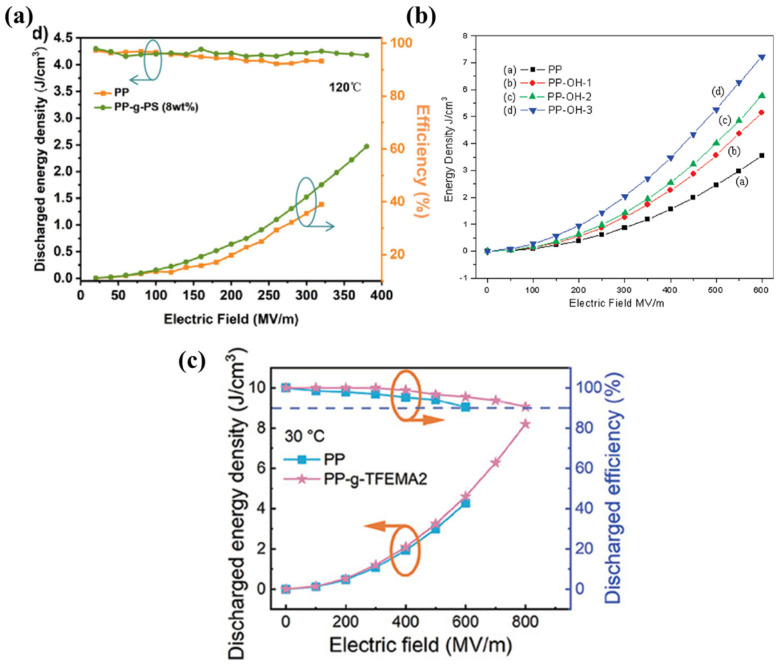
(**a**) Energy density and charge/discharge curves of PP-g-PS at 120 °C [99]. Copyright 2025, ACS *Applied Polymer Materials*. (**b**) Energy density curves of hydroxylated BOPP vs. unhydroxylated BOPP. PP-OH-1 is the 0.7 mol of OH copolymer monomer unit, PP-OH-2 is the 1.8 mol of OH copolymer monomer units, PP-OH-3 is the 4.2 mol of OH copolymer monomer units [100]. Copyright 2010, *Macromolecules*. (**c**) Energy density and charge/discharge curves of TFEMA-g-PP at 30 °C [101]. Copyright 2024, *Advanced Functional Materials*.

The main chain of PP is composed entirely of a carbon–carbon structure without any polar groups, which makes its dielectric constant low, seriously limiting improvements in the energy storage density of BOPP and the ability to meet the needs of modern electrical equipment [35,96]. Styrene (PS) has a certain degree of rigidity due to its own benzene ring structure; grafting it to PP is conducive to the formation of more nucleation sites, and the formation of more interfacial regions increases the interfacial polarization and can also introduce deeper traps to capture more carriers, reducing carrier mobility [97,98]. Furthermore, electron beam radiation grafting has been shown to be an effective method for preventing the entry of impurities without the need for additional additives. Concurrently, the grafting side of the electron beam radiation can introduce greater numbers of oxygen-containing functional groups, thereby increasing the polarity of the PP. In addition, the free radicals generated can form new traps to capture more carriers. The grafted film has an energy storage density of up to 5.43 J cm^−3^ at room temperature, and even at 110 °C, it is maintained at 3.44 J cm^−3^, compared to that of the pure PP of only 1.78 J cm^−3^, as shown in Figure 11a. This is attributed to the deeper trap energy level and higher trap density of the grafted PP [99]. Hydroxyl(-OH) is the most common polar group; grafting it to PP results in a more favorable enhancement of the energy storage density. The energy density of 7.42 J cm^−3^ at a field strength of 600 MV m^−1^ is more than twice that of unhydroxylated BOPP, as shown in Figure 11b below [100]. Trifluoroethyl fluoromethacrylate (TFEMA) with strong polarity was grafted onto PP, and it enhanced the latter’s breakdown strength to 865 MV m^−1^ while maintaining an energy density of 8.2 J cm^−3^ and charging and discharging efficiencies of more than 90%; the energy density is shown in Figure 11c [101]. This may be due to the fact that the graft-modified PP induces the generation of an α-crystalline type with smaller and denser grains and higher crystallinity, and also because the higher crystallinity enhances the mechanical properties, according to the Stark–Garton equation $E_{b}=0.606{\left(Y/\left(\varepsilon_{0}\varepsilon_{r}\right)\right)}^{0.5}$, where Y stands for the Young’s modulus [102]. The enhanced mechanical properties of the films are positively correlated with enhanced breakdown performance. TFEMA significantly enhances the charge trapping ability of electrons and holes, while the formation of additional micro-interfaces between the small-sized crystal particles inhibits the migration of injected charges at high electric fields.

Graft modification enhances the dielectric stability and energy storage density of PP while exhibiting flexibility. It does not diminish PP’s insulation properties. The presence of polar groups enhances the dielectric constant and constricts molecular movement. However, this process also introduces deep traps, which may lead to an uneven electrical field distribution. The grafting group does not guarantee a high grafting rate, creating a new problem: what should be done with the “impurities” that are not grafted on. The grafting method needs improvement, as do the selection, dosage, and method of grafting groups, to meet the requirements of high-performance PP.

### 2.4. Multilayer Structure Design

**Figure 12:**
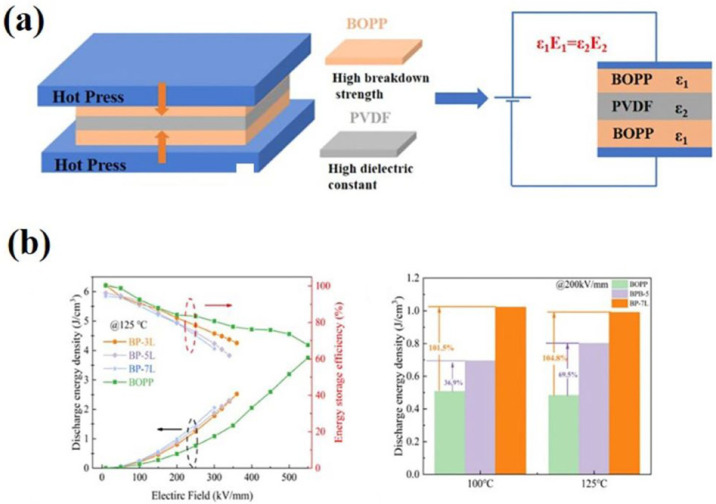
(**a**) Process flow of multilayer structure construction with PVDF as the middle layer, flanked by BOPP. (**b**) Storage performance of multilayer composite film at 125 °C and discharge energy density at high temperature: BP-3L represents a 3-layer structure, BP-5L represents a 5-layer structure, BP-7L represents a 7-layer structure [104].

**Figure 13:**
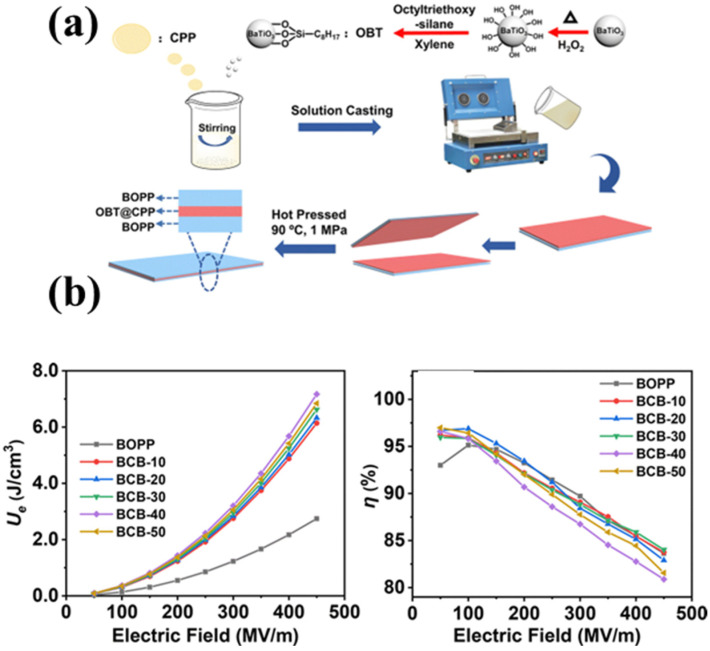
(**a**) Process flow diagram of BOPP-OBT@CPP-BOPP composite film. (**b**) Energy storage density and charge/discharge efficiency of the composite film; BCB-10 represents a 10-layer structure, BCB-20 represents a 20-layer structure, BCB-30 represents a 30-layer structure, BCB-40 represents a 40-layer structure, BCB-50 represents a 50-layer structure [105]. Copyright 2022, *Journal of Materials Chemistry C*.

The design of a multilayer structure can effectively utilize the advantages of different layers to reduce the energy loss, and the interfacial effect generated between layers can effectively inhibit the carrier transport, which in turn improves the dielectric energy storage capacity of BOPP. The C-F bond in the main chain of polyvinylidene fluoride (PVDF) has a high dipole moment and stacking density, and thus its dielectric constant is also very large at 10–50 [103]; the use of other high dielectric properties can improve the energy storage performance of BOPP at high temperatures. By constructing a multilayer structure with PVDF as the middle layer flanked by BOPP, the energy storage performance of BOPP under high temperatures and low electric fields can be effectively enhanced; the preparation method is shown in Figure 12a. When the number of constructed layers is 7, the energy storage density at 125 °C and 200 MV m^−1^ is increased by 104.8% compared with that of pure PP, as shown in Figure 12b [104]. Researchers have been searching for a way to incorporate the high dielectric constant of ferroelectric ceramic particles into BOPP in a reasonable manner; for this, they designed a multilayer structure with an intermediate layer of octyltriethoxysilane (OBT)-modifiedBaTiO_3_ as a high-dielectric filler blended with chlorinated polypropylene (CPP), and the homogeneously dispersed modified BaTiO_3_ in the polarized CPP further enhanced the dielectric constant, while the outer layer of BOPP provided effective electrical insulation and excellent breakdown strength. The preparation process is shown in Figure 13a. This produced a composite film with an energy density of up to 7.17 J cm^−3^ and an energy storage density 2.6 times that of ordinary BOPP, as shown in Figure 13b. This is attributed to the strong polarity of the middle layer and the high insulation of the outer layer [105]. The same PANI-coated BaTiO_3_ as filler was blended with CPP to form the intermediate layer, and the modified multilayer structured film had an energy storage density of 7.31 J cm^−3^ at a field strength of 450 MV m^−1^, which was attributed to the mulberry-like BaTiO_3_@PANI, which isolated the conductive paths as well as the core–shell BaTiO_3_@PANI, which could inhibit distortions under high electrical fields [106]. Boron nitride sheet (BNNSs) is a commonly used ceramic material with a high dielectric constant, and it was synthesized into a core–shell structure of BNNSs@PDA using polydopamine (PDA) as a modified shell to improve the dispersion and compatibility of the filler in PP, with BNNSs/PP as the outer structure, while the interlayer was Ba_0.7_Sr_0.3_TiO_3_@Polydopamine (BST@PDA/PP). In terms of performance, its breakdown strength and energy storage density were 39.38% and 189.29% higher than those of PP, at 325 MV m^−1^ and 1.96 J cm^−3^, respectively [107]. This is because the BNNSs/PP in the outer layer effectively enhanced the breakdown strength of the composite film due to the presence of BNNSs with a wide bandgap, while the BST@PDA in the middle layer increased the overall polarizability of the film with a higher dielectric constant. In addition, the PDA modification increased the compatibility and prevented the deterioration of the properties due to the localized electrical field concentration and reduced the concentration of charge on the filler surface, inhibiting the charge migration at the interface.

In designing multilayer structures, it is crucial to prioritize quality interlayer bonding. Optimal interfacial bonding prevents defect formation and impedes charge transportation. Poor interlayer bonding can compromise performance and safety. Each layer must be meticulously prepared to minimize defects. Future research should focus on improving layer-to-layer bonding, optimizing single-layer performance, and understanding how multilayer structures store energy.

## 3. Processing Affects Polypropylene Energy Storage and Dielectric Properties

PP’s processing and stretching can also impact performance. High temperatures during processing deteriorate the film, preventing optimal performance. The biaxial stretching process is important, as it has been shown to improve the film’s mechanical and dielectric properties. However, defects are introduced during stretching due to various factors. Despite the lack of research in this area, efforts are underway to improve the stretching process and minimize defect introduction. These efforts focus on optimizing BOPP’s performance to address a key research gap.

### 3.1. Processing Technology

Annealing is an important process for changing the properties of plastics. During the annealing process, the relaxation of the oriented chains is accompanied by the rearrangement of the molecular chains, which reduces the defects in the crystals and increases their orientation, leading to changes in the internal structure and properties. Annealing increases the diffusive motion of the molecular chains, which leads to an increase in the thickness of the internal lamellar crystals, which contributes to the homogenization of the film thickness [108]. This leads to the construction of a more homogeneous crystal network structure of reinforcing fibers, a phenomenon that helps to reduce the generation of BOPP defects and improve the BOPP’s electrical insulation properties and dielectric constant. However, with an increase in annealing temperature, the breakdown strength of BOPP showed a significant deterioration, unlike in the unidirectional stretching of PP, where it first increases and then decreases, which may be due to the severe destruction of the orientation of BOPP at high temperatures [109]. Inorganic fillers tend to show unsatisfactory performance enhancement or even huge performance defects due to agglomeration problems; to ameliorate this problem, the dispersion of fillers was improved through high-shear extrusion, and the incorporation of montmorillonite (oMMT) and BNNs provided the PP with reliable dielectric properties, leading to an increase in energy storage density from 2.98 J cm^−3^ to 5.14 J cm^−3^. This can be attributed to the fact that the high shear force applied to the nanofillers greatly alleviated the van der Waals interaction forces between the filler particles, making the blending more homogeneous [110]. Graphene nanoparticles (GNPs) are also currently a popular filler with researchers; however, the agglomeration problem can also limit the enhancement in PP performance. Therefore, the dispersion within PP was improved using a high-shear extrusion technique, and the relative permittivity was consequently increased from 2.39 to 2.66 [111]. This was due to the fact that, with an increase in the shear rate, the size of the agglomerates was effectively reduced, which resulted in a more homogeneous blend. The size was effectively reduced, and fewer interfacial defects were created between the filler and the matrix, introducing more charge traps and limiting the movement of carriers.

Processing technology is an important part of plastic molding. The process will have a significant impact on the subsequent stretching process and determines the quality of the final product produced. Therefore, each link of the process should be explored in depth; at the same time, it is necessary to clarify the mechanisms by which they impact PP and continuously optimize and improve the processing parameters and procedures.

### 3.2. Stretching Technique

**Figure 14:**
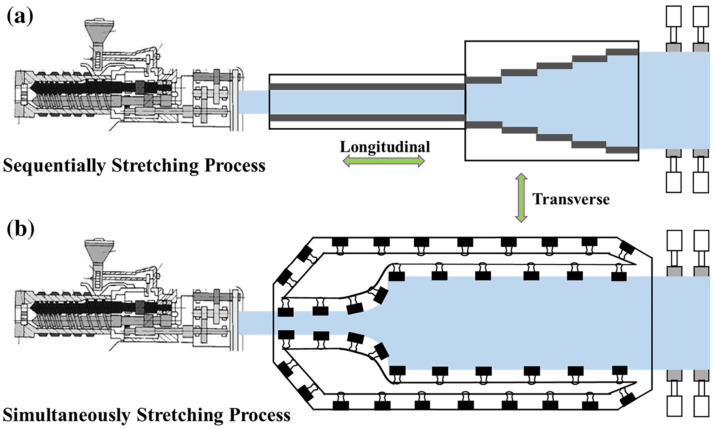
BOPP biaxial stretching process: (**a**) sequential biaxial stretching; (**b**) synchronous biaxial stretching [109]. Copyright 2020, *Journal of Applied Polymer Science*. The BOPP stretching link is the last and most important link; the quality of the film’s stretching directly determines the product’s quality. It is necessary to further explore the stretching process; different raw materials need to be used in different stretching modes, the defects in the stretching process need to be minimized, and a clear stretching mechanism to optimize the stretching parameters needs to be developed.

Bi-directional stretching is necessary for the preparation of PP films for capacitors. Compared with the conventional cast film making method, bi-directional stretching can make the molecular chain arrangement more orderly, the crystalline region more dense, and the orientation more complete [29], thus inhibiting the carrier mobility in the amorphous region. In commerce, there are currently two main stretching methods used to produce BOPP: sequential and co-bi-directional. These two stretching processes are shown in Figure 14. Sequential stretching results in an anisotropic character of the PP’s internal crystals in both directions, while synchronous stretching is isotropic [110,112]. Upon comparing the two different stretching modes, it is found that synchronous biaxial stretching results in superior crystallinity, more homogeneous crystal dispersion, and a higher breakdown strength and energy storage density [111]. This is because synchronized stretching introduces fewer defects and produces superior stretching uniformity. In addition, the control of the temperature range during bi-directional stretching also has an effect on the BOPP, and the uniformity of the BOPP’s thickness is improved when the stretching temperature region is properly expanded [112]. This is because, for low-melting-point components, low-temperature stretching contributes to a higher degree of orientation; in addition, an increase in the stretching temperature can lead to an increase in the film’s tensile ratio and a decrease in thickness. For sequential bi-directional stretching, the effect of processing temperature occurs mainly during transverse stretching [110]. The stretching process also has different effects on different feedstocks; LCBPP has more long branched structures than PP, which effectively allow the molecular chains to become entangled to varying degrees, thus inhibiting the movement of the molecular chains at elevated temperatures. However, these long branched structures also reduce the regularity of the molecular chains, leading to a decrease in crystallinity, which in turn affects the dielectric and mechanical properties of BOPP. Through biaxial stretching and comparing LCBPP and PP, it is proved that the stretching process can effectively enhance the dielectric properties at high temperatures The breakdown strength at 125 °C is 606.9 MV m^−1^ [110]. However, the enhancement effect of biaxial stretching for LCBPP is significantly larger than that for PP, which may be due to the fact that the crystallinity of PP itself is relatively high, and the bi-directional stretching affects the crystal’s orientation more than it affects the crystallinity, whereas for LCBPP, both the orientation and crystallinity are enhanced, and thus the enhancement effect of bi-directional stretching is more significant.

## 4. Outlook and Perspective

This paper offers methodologies for enhancing the dielectric stability of PP at elevated temperatures, increasing the energy storage density and efficiency in production. It also describes stretching process methodologies for improving performance. These methodologies are expected to serve as a valuable reference for developing high-performance PP. A synopsis of this study is provided:

(1) The research on PP with high dielectric stability and high energy storage density focuses on blending and doping, as well as new molecular structures. Meanwhile, the research on surface modification and multilayer structures is limited. This is due to the efficacy and expediency of co-doping and its more discernible mechanism. This research used ceramic materials with wide bandgaps, harnessing the interfacial effect of nano-fillers to create extra traps that capture charges, which hinders the injection and transport of carriers and the formation of electrical trees. This improves the electrical insulation and energy storage density. However, the compatibility of fillers with PP remains to be resolved. The creation of new molecular structures involves the grafting of polar groups and the establishment of crosslinked structures. Deep traps within the molecular chain limit carriers’ movement, while a crosslinked structure restricts molecular chains’ movement at elevated temperatures. Polar molecules enhance the polarity of the PP and its dielectric constant. However, its industrial application is limited by the complex modification process. Although the design of a multi-layer structure can effectively improve the energy storage of BOPP, there are still interface problems between the layers. Although surface modification has great potential for development, there are still problems with complex processes and mediocre effects in the existing literature. Simple filler modification is convenient, fast, and more suitable for industrial production, while the construction and design of new molecular structures, surface modification, and the design of multi-layer structures are relatively complex in operation and principle, making them more suitable for scientific research and exploration.

(2) Although the effects of PP processing and stretching on the finished product are well-known, there has been little research into their effects. This is mainly due to the fact that these processes do not have significant effects on the properties of PP and the complexity of the research process. As a result, research has primarily focused on PP materials. There is a wide range of PP materials on the market, and different processes have different effects on each material. This makes it difficult to establish general principles that can effectively guide the study of processing and stretching processes. The main focus regarding the processing is investigating how to improve the agglomeration of fillers in BOPP and the effect on the molecular chain and crystallinity of BOPP, while the stretching process mainly affects the crystallization and molecular chain orientation of BOPP.

Although significant progress has been made in the research to achieve polypropylene with high dielectric stability and high energy storage density, there is still room for improvement in the current research: the performance of BOPP decreases significantly at high temperatures, the energy storage density of BOPP is still too low compared to electrochemical principles, and commercial production problems remain. These issues mean that current dielectric capacitors cannot meet the requirements of miniaturization and integration in electronic devices. Overcoming these obstacles is crucial for further research on polypropylene with high dielectric stability and high energy storage density, which is of great significance for the updating and iteration of electronic devices such as mobile phones, wearable devices, and electronic cars. The following research approach is expected to address these challenges. (1) For filler modification, the investigation of filler and PP dispersion should be further developed. This will improve the dielectric properties and thermal stability of PP after the introduction of interfacial effects and reduce the generation of interfacial defects. The advantages of the co-dopant should be fully harnessed while avoiding defects caused by it. Secondly, the choice of appropriate crosslinking or grafting methods is crucial in creating traps and limiting the movement of molecular chains when constructing new molecular structures. The introduction of impurities should be minimized during or after the modification process. This will prevent them from affecting the PP’s properties. We should continue to refine and improve crosslinking and grafting methods to enhance the grafting rate and crosslinking degree. It is important to ensure the long-term reliability and safety of the modified PP. At the same time, when using surface modification methods to improve the energy storage performance of BOPP, it is necessary to simplify the operation as much as possible and further clarify the surface modification mechanism. In addition, simplifying the process flow for designing multilayer structures and reducing the multilayer composite film thickness are also recommended. Optimization of the preparation process for the aforementioned modification methods is advised to make the process easier and improve the operability of industrial production.

(2) We should continue to study the processing and stretching process and clarify the processing’s impact on the general mechanism of PP, especially exploring the impacts of the preparation process parameters and optimizing them. It is necessary to reduce the defects of BOPP in the manufacturing process. Efforts should be made to improve and compensate for the deficiencies and defects in the modification process through processing and stretching techniques, in order to reduce the damage to BOPP during the process.

## Data Availability

No new data were created or analyzed in this study. Data sharing is not applicable to this article.
